# Trochanteric Buttress Plate Augmentation of the Proximal Femoral Nail for Unstable Intertrochanteric Fractures: A Randomized Controlled Trial

**DOI:** 10.7759/cureus.104235

**Published:** 2026-02-25

**Authors:** Mohamed H Abdelaty Shalaby, Doaa G Khafagy, Mohamed M Hosny, Ahmed E Eltantawy, Ali M Omran

**Affiliations:** 1 Department of Trauma and Orthopaedic Surgery, The Shrewsbury and Telford NHS Trust, Telford, GBR; 2 Department of Acute Medicine, The Shrewsbury and Telford NHS Trust, Telford, GBR; 3 Department of Trauma and Orthopaedic Surgery, Faculty of Medicine, Tanta University, Tanta, EGY

**Keywords:** fracture fixation, intertrochanteric fracture, modified harris hip score, proximal femoral nail, trochanteric buttress plate

## Abstract

Background: Unstable intertrochanteric (IT) fractures pose a significant challenge in orthopedic surgery.

Objective: To investigate the effectiveness of combining a trochanteric buttress plate (TBP) with a proximal femoral nail (PFN) for managing unstable IT fractures.

Methods: This randomized controlled open-label study was performed on 90 patients with recent unstable IT fractures (Evans types 1c, 1d, 1e, or 2). Patients were randomly divided into two groups: Group A was treated with standard PFN fixation. Group B was treated with PFN integrated with TBP augmentation. Assessments were conducted at two weeks and monthly for six months. Functional outcomes were assessed using the modified Harris Hip Score (mHHS).

Results: Operative time and blood loss were significantly higher in Group B (PFN+TBP) than in Group A (standard PFN) (P = 0.005 and 0.006, respectively). Postoperative mHHS was significantly higher in Group B at all follow-up intervals from two weeks to six months (P < 0.05).

Conclusions: The integration of the TBP with PFN for unstable IT fractures significantly improves functional outcomes without increasing major complications, despite a modest increase in operative time and blood loss.

## Introduction

Intertrochanteric (IT) fractures are among the most common hip fractures, particularly in the elderly, accounting for approximately 50-60% of all hip fractures. These fractures often occur due to low-energy falls, especially in individuals with osteoporosis and frailty, which makes their bones more susceptible to fracture even with minimal trauma. Osteoporosis, characterized by reduced bone density, is a major risk factor for these fractures. Additionally, aging and muscle weakness (sarcopenia) significantly increase the likelihood of falls and fractures [1]. These fractures occur between the greater and lesser trochanters and are classified as stable or unstable based on the degree of fragmentation [2].

Unstable IT fractures are characterized by significant comminution, loss of medial support, and a breach in the lateral wall cortex [3]. They remain associated with high complication rates, posing a significant public health and economic burden [4].

Despite advancements, the optimal management of unstable IT fractures is debated [5]. Intramedullary nails, like the proximal femoral nail (PFN), are effective for stabilization [6]. Mehta et al. reported excellent long-term functional outcomes with PFN for IT fractures [7].

The trochanteric buttress plate (TBP) is an implant fixed to the lateral femur designed to provide supplemental support, reducing the risk of displacement and collapse [8]. The integration of TBP with PFN involves fixing the plate to the lateral femur and connecting it to the intramedullary nail, creating a stable construct that resists rotational and bending forces [8-10].

The TBP is primarily designed to provide resistance to bending forces by supporting the lateral wall of the proximal femur. While it can contribute to stability under certain rotational forces, true rotational control is more commonly achieved with anterior or anti-rotation plating techniques. More specific biomechanical evidence is required to fully support its role in resisting rotational forces, as most studies focus on its effectiveness in lateral wall support and bending force resistance [11].

Devices placed outside the medullary canal offer a different approach to proximal femoral nails (PFN). While proximal femoral plates provide excellent fixation and are often used in more complex fractures, PFN is preferred in many clinical scenarios due to its minimally invasive nature and ability to achieve sufficient stabilization with reduced surgical morbidity. There is a lack of randomized controlled trials comparing the two methods in terms of healing, functional recovery, complications, biomechanical benefits, and cost-effectiveness. Thus, this study aimed to compare the functional outcomes, radiological results, and complication rates between two surgical techniques for treating unstable intertrochanteric fractures: PFN and TBP augmentation.

The hypothesis behind our study is that the PFN combined with the TBP provides superior stability and better functional outcomes compared to traditional fixation methods, particularly in unstable intertrochanteric fractures.

## Materials and methods

This randomized controlled open-label study was conducted between October 2022 and September 2023 after approval from the Ethical Committee of Tanta University Hospitals, Tanta, Egypt (approval code: 35951/10/22, dated October 12, 2022) and registration of Pan African Clinical Trials Registry (PACTR) (PACTR202512641559762). All patients provided informed written consent.

We included 90 patients of both sexes with recent traumatic, isolated, unstable IT fractures (Evans type 1c, 1d, 1e, or 2). Exclusion criteria were neurological deficit, uncontrolled comorbidities, open fractures, proximal femoral deformity, pathological fractures, and previous ipsilateral femoral surgery.

All patients underwent history taking, clinical examination, laboratory tests, and radiological investigations (plain X-rays and CT if needed).

Randomization

Patients were randomly allocated using an online randomizer (www.randomizer.org) into two equal groups: group A (n=45): standard PFN fixation; group B (n=45): PFN integrated with TBP augmentation.

The study was open-label due to the nature of the interventions.

Surgical technique

All procedures were performed under spinal or general anesthesia. Patients were positioned supine on a traction table, and fracture reduction was achieved through longitudinal traction and internal rotation under C-arm fluoroscopic guidance. Where necessary, temporary fixation was achieved with a 3 mm K-wire to maintain reduction.

A standard technique was used for the insertion of the PFN. An entry point was established, the canal was prepared, and for short nails an appropriately sized nail (18 cm or 24 cm) was inserted, and for long nails the size was measured, and variable sizes were available (34 to 40).

Trigen Intertan (Smith & Nephew, Memphis, TN, USA) or Gamma nails (Stryker, Kalamazoo, MI, USA) were used for both groups. This is a locking intramedullary nail designed for the treatment of femoral fractures. It provides excellent support and stability, with distal interlocking screws used for fixation.

The TBP was used for Group B. This lateral compression plate is specifically designed to augment the stabilization provided by the PFN.

TBP augmentation

In the study group (Group B), the TBP was applied following nail insertion. The plate was introduced through the existing proximal incision. After gentle subperiosteal dissection to expose the trochanteric flare, the plate was positioned over the lateral wall.

The key holes of the TBP were aligned with the corresponding screw holes in the proximal nail. Using the Trigen Intertan, the lag and derotation screws were then inserted through the plate into the femoral head and neck, thereby interconnecting the two implants. In the Gamma nail, the lag screw was introduced first before the plate, then the plate was inserted. Following this, the anti-rotation screw was introduced, compressing the plate. Finally, the distal portion of the plate was secured to the femoral shaft with unicortical or bicortical screws through its inferior holes, completing the stable, combined construct. There are also proximal holes for more cortical screws, if needed, around the greater trochanter area.

A vacuum drain was used at the surgeon's discretion. Intraoperative data, including operative time and blood loss, were recorded.

The TBP used was manufactured by Smart Tech Solutions, Egypt, supplied by Almasrya Medical Company and Elshamy Orthopedic Company, Egypt. The plate is a non-locking stainless steel plate. The plates are available in lengths ranging from three to seven distal shaft holes, depending on the length required, allowing the surgeon to select the most appropriate size based on the fracture location and patient anatomy. The proximal or distal non-locking screws used were 4.5 mm.

Postoperative protocol and outcome measures

Following surgery with a PFN and TBP, weight-bearing instructions typically vary based on fracture stability and healing progress. In the immediate postoperative period (0-2 weeks), patients are usually advised to be non-weight-bearing (NWB) or partial weight-bearing (PWB), using crutches or a walker. After two to six weeks, partial weight-bearing is generally permitted as the fracture begins to stabilize. Full weight-bearing is typically allowed after six weeks, depending on radiographic confirmation of healing. However, weight-bearing instructions should always be personalized by the surgeon based on the patient’s specific condition and progress.

In our study, we assessed the quality of reduction at multiple follow-up points, including immediately postoperatively, at three months, and at six months. Initially, the reduction was evaluated based on radiographic alignment and the proper positioning of the fixation devices. Over the course of the following months, we observed potential changes in alignment or fixation, such as malunion, loss of reduction, or screw loosening, which could affect the overall quality of the reduction. At the six-month follow-up, we looked for any signs of fracture healing or complications that might indicate a shift in the quality of reduction. In some cases, initial satisfactory reduction may have been maintained, while in others, slight adjustments or complications arose as healing progressed, which we documented. These findings were then considered when evaluating long-term outcomes and the success of the procedure.

**Figure 1:**
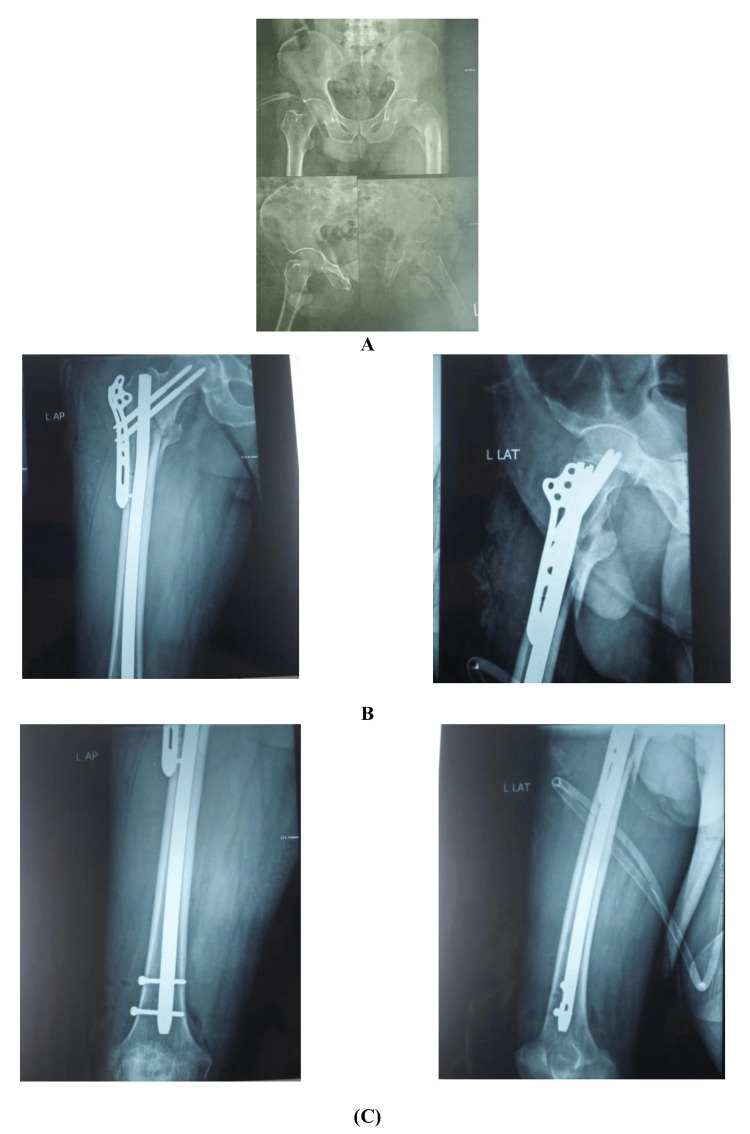
Usage of long nail (A) Preoperative X-rays show an unstable inter-trochanteric femur fracture. (B) Postoperative anteroposterior X-ray shows a nicely reduced fracture. (C) Anteroposterior postoperative X-ray shows the length of the nail, and the lateral postoperative X-ray shows the length of the nail.

**Figure 2:**
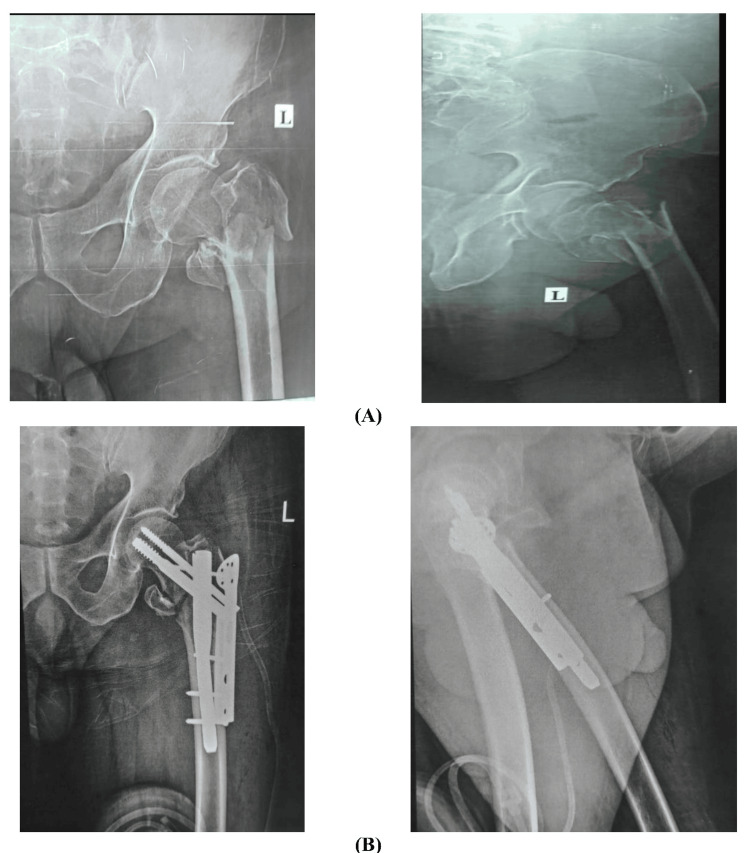
Short nail usage (A) Preoperative X-rays show an unstable inter-trochanteric femur fracture. (B) Postoperative anteroposterior and lateral X-ray shows a nicely reduced fracture.

Follow-up assessments were conducted at two weeks and then monthly for six months. Clinical and radiological evaluations were performed at each visit. The primary functional outcome was measured using the modified Harris Hip Score (mHHS) [11,12]. Radiographic outcome was assessed by evaluating the quality of fracture reduction and union on standard anteroposterior and lateral radiographs. All complications were recorded (Figures 1, 2).

Assessment of fracture reduction quality

Fracture reduction quality was assessed on immediate postoperative anteroposterior and lateral radiographs using validated criteria adapted from Baumgaertner et al. and Fogagnolo et al. [13,14].

Reduction was graded as good, fair, and poor.

Good

All of the following criteria met: neck-shaft angle 125°-135° (anatomic or slight valgus); angulation <10° on both AP and lateral views; cortical apposition with displacement <2 mm; tip-apex distance (TAD) <25 mm; lag screw positioned in center-center or inferior-center quadrant.

Fair

One or two of the following met: mild varus (≤5°) or valgus (135°-140°); cortical displacement 2-5 mm; TAD 25-30 mm; screw in anterior or posterior quadrant.

Poor

Any of the following met: varus >5° or valgus >140°; cortical displacement >5 mm; TAD >30 mm; screw penetration or superior cut-out position; any rotational malalignment.

Primary and secondary outcomes

The primary outcome was functional recovery assessed using the modified Harris Hip Score (mHHS).

The preoperative mHHS was not measured in the acute post-injury setting. Instead, patients were interviewed at the time of admission and asked to recall their functional status during the week immediately preceding the fracture. This methodology is standard in hip fracture research when prospective pre-injury baseline data cannot be obtained [11,15].

Postoperative mHHS was assessed at two weeks and then monthly for six months based on the patient's current functional status at each visit.

Secondary outcomes included: operative time (minutes), intraoperative blood loss (mL), need for blood transfusion, time to radiographic union (weeks), quality of fracture reduction, re-operation for mechanical failure, and complications (cut-out, varus collapse, implant failure, delayed union, infection, lateral thigh pain).

Sample size calculation

Using G*Power 3.1.9.2 (Heinrich-Heine-Universität Düsseldorf, Düsseldorf, Germany), with α=0.05 and power=80% to detect a 30% increase in excellent mHHS (from 20% to 50%), a sample of 39 per group was needed. We recruited 45 per group to account for dropouts.

Statistical analysis

Data were analyzed using IBM SPSS Statistics for Windows, version 26 (IBM Corp., Armonk, NY, USA). Normality was assessed with the Shapiro-Wilk test. Quantitative data (mean ± SD) were compared with T-tests. Qualitative data (frequency, %) were compared with Chi-square tests. A P-value ≤ 0.05 was considered significant.

## Results

**Figure 3:**
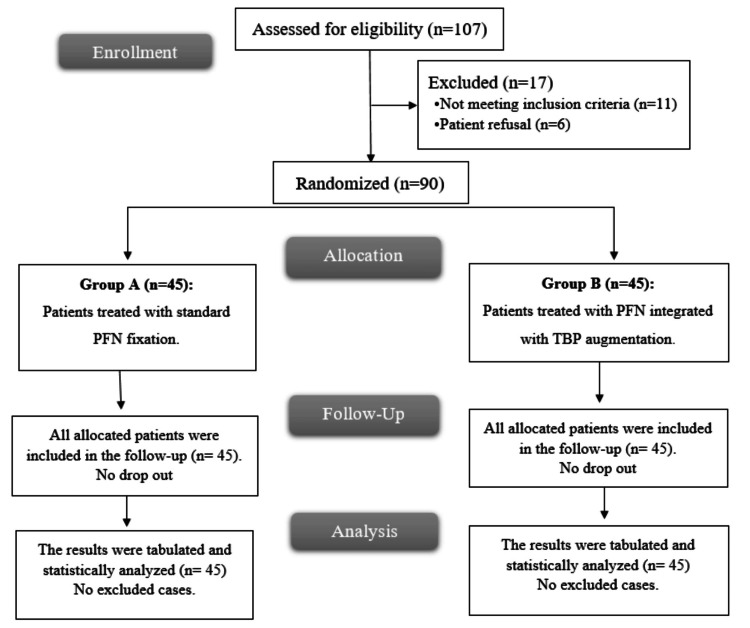
CONSORT flow chart of the enrolled patients CONSORT: Consolidated Standards of Reporting Trials

A total of 107 patients were assessed for eligibility, of whom 17 were excluded (11 did not meet the inclusion criteria and six declined to participate). Ninety patients were subsequently randomized into two equal groups. Group A included 45 patients who underwent standard PFN fixation, while group B included 45 patients treated with PFN integrated with TBP augmentation. All allocated patients in both groups completed the follow-up period with no dropouts. The outcomes of all enrolled patients (n = 45 in each group) were included in the final statistical analysis, with no excluded cases (Figure 3).

Age, sex, weight, height, BMI, smoking, and comorbidities were not significantly different between the two groups (Table 1).

**Table 1:** Demographic data and risk factors of the studied groups

Parameter			Group A (n=45)	Group B (n=45)	P	Mean difference or RR (95% CI)
Age (years)			55.18 ± 8.44	54.73 ± 7.59	0.794	0.44 (-2.92:3.8)
Sex		Male	28 (62.22%)	32 (71.11%)	0.668	0.88 (1.17-0.65)
		Female	17 (37.78%)	13 (28.89%)		
Weight (kg)			89.69±12.53	86.96±12.59	0.305	2.7 (-2.53:7.99)
Height (cm)			172.62 ± 5.98	171.22 ± 3.3	0.172	1.4 (-0.62:3.42)
BMI (kg/m^2^)			30.13 ± 4.22	29.69 ± 4.39	0.629	0.44 (-1.36:2.4)
Smoking			12 (26.67%)	16 (35.56%)	0.362	0.75 (0.4:1.4)
Comorbidities	Diabetes mellitus		14 (31.11%)	16 (35.56%)	0.655	0.88 (1.57:0.49)
	Hypertension		12 (26.67%)	10 (22.22%)	0.624	1.2 (2.49-0.58)
	Immunological disorders		2 (4.44%)	1 (2.22%)	0.557	2 (0.19:21.28)

The side of injury, need for blood transfusion, time to radiographic union, and re-operation for mechanical failure were not significantly different between the two groups. Operative time and blood loss were significantly higher in group B than in group A (P value=0.005 and 0.006, respectively) (Table 2).

**Table 2:** Intraoperative data, time to radiographic union need of blood transfusion and re-operation for mechanical failure of the studied groups

Parameter		Group A (n=45)	Group B (n=45)	P	Mean difference or RR (95% CI)
Side of injury	Right	26 (57.78%)	31 (68.89%)	0.274	0.84 (1.15:0.61)
	Left	17 (37.78%)	13 (28.89%)		
Operative time (min)		76.22 ± 19.07	87.33 ± 17.82	0.005	-11.1 (-18.8: -3.4)
Blood loss (ml)		156.09 ± 46.32	191.49 ± 69.92	0.006	-0.35.4 (-60.25: -10.55)
Need for blood transfusion		2 (4.44%)	4 (8.89%)	0.398	0.5 (0.1:2.59)
Time to radiographic union (week)		14.56 ± 4.21	13.44 ± 3.86	0.195	1.11 (-0.58:2.8)
Re-operation for mechanical failure		2 (4.44%)	1 (2.22%)	1	2 (21.28:0.19)

Preoperative mHHS was not significantly different between the two groups. Postoperative mHHS was significantly higher in group B than group A at 2 w, 1 m, 2 m, 3 m, 4 m, 5 m, and 6 m (P value<0.05) (Table 3).

**Table 3:** Modified Harris hip score of the studied patients

Parameter		Group A (n=45)	Group B (n=45)	P	Mean difference (95% CI)
Preoperative		27.8±6.87	25.4±7.97	0.130	2.4 (-0.71:5.5)
Postoperative	2 w	36.2±7.64	39.58±7.91	0.042	-3.4 (-6.6: -0.11)
	1 m	38.73±7.37	46.78±7.76	<0.001	-8.04 (-11.2: -4.8)
	2 m	44.96±7.38	53.27±7.74	<0.001	-8.3 (-11.4: -5.14)
	3 m	55.89±7.49	64.13±8.24	<0.001	-8.2(-11.5: -4.94)
	4 m	68.82±8.2	72.76±8.21	0.025	-3.9 (-7.3: -0.49)
	5 m	78.24±8.17	82.8±7.64	0.008	-4.5 (-7.87: -1.24)
	6 m	80.11±8.16	84.91±7.49	0.005	-4.8 (-8.08: -1.51)

The quality of reduction was not significantly different between the two groups at all measurements (Table 4).

**Table 4:** Quality of reduction of the studied groups

Parameter		Group A (n=45)	Group B (n=45)	P
2 w	Good	14 (31.11)	15 (33.33%)	0.892
	Fair	18 (40)	19 (42. 22%)	
	Poor	13 (28.89)	11 (24.44%)	
1 m	Good	20 (44.44%)	26 (57.78%)	0.435
	Fair	18 (40%)	13 (28.89%)	
	Poor	7 (15.56%)	6 (13.33%)	
2 m	Good	28 (62.22%)	34 (75.56%)	0.301
	Fair	10 (22.22%)	8 (17.78%)	
	Poor	7 (15.56%)	3 (6.67%)	
3 m	Good	32 (71.11%)	39 (86.67%)	0.101
	Fair	7 (15.56%)	5 (11.11%)	
	Poor	6 (13.33%)	1 (2.22%)	
4 m	Good	37 (82.22%)	41 (91.11%)	0.463
	Fair	6 (13.33%)	3 (6.67%)	
	Poor	2 (4.44%)	1 (2.22%)	
5 m	Good	39 (86.67%)	43 (95.56%)	0.312
	Fair	4 (8.89%)	1 (2.22%)	
	Poor	2 (4.44%)	1 (2.22%)	
6 m	Good	42 (93.33%)	44 (97.78%)	0.501
	Fair	1 (2.22%)	0 (0%)	
	Poor	2 (4.44%)	1 (2.22%)	

**Figure 4:**
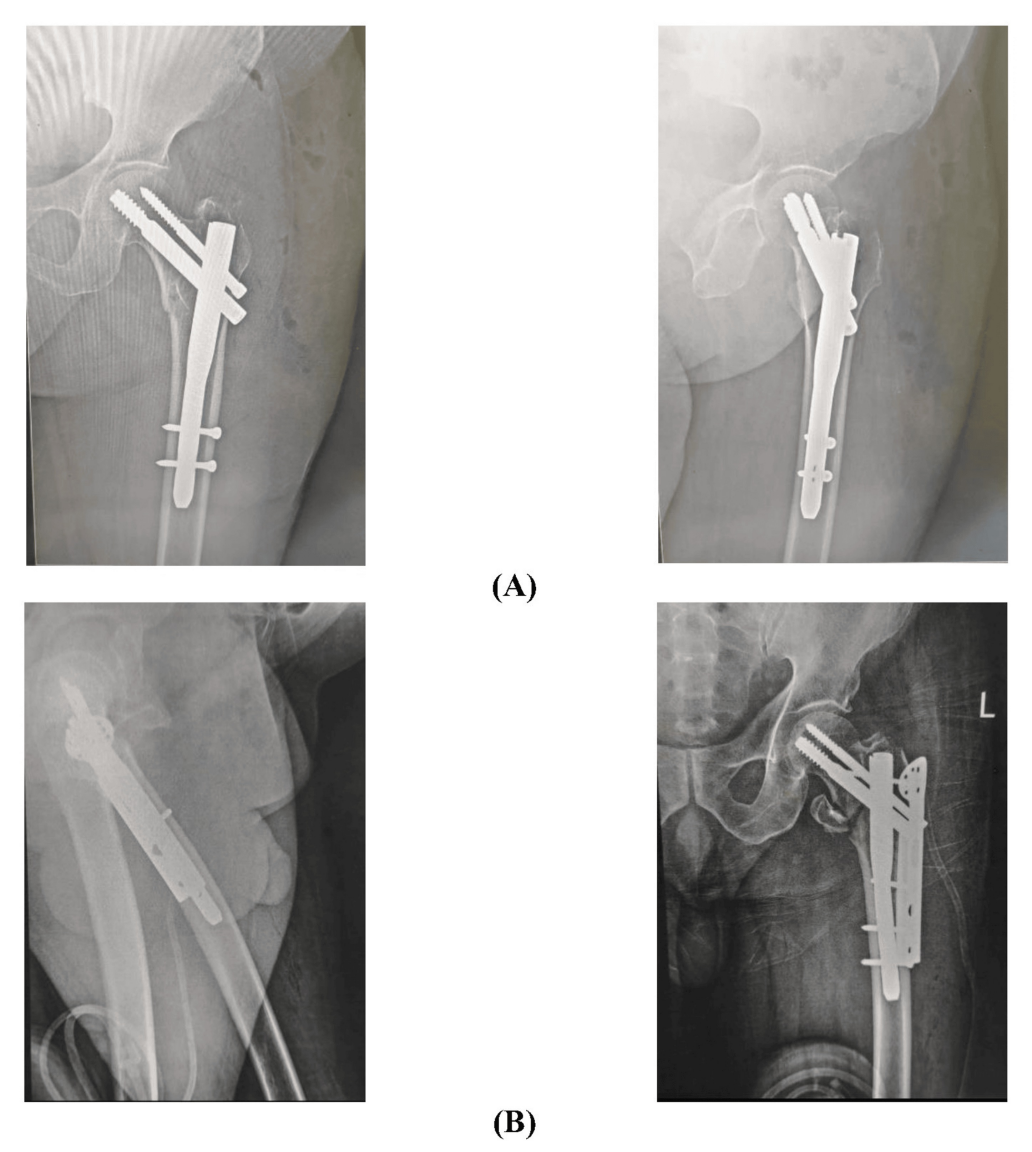
(A) Postoperative X-rays showing intertrochanteric femur fracture managed by PFN only, and (B) postoperative X-rays showing intertrochanteric femur fracture managed by PFN with TBP augmentation PFN: proximal femoral nail; TBP: trochanteric buttress plate

Reduction quality was graded as good, fair, or poor based on predefined radiographic criteria, including neck-shaft angle, angulation, cortical displacement, tip-apex distance, and screw position. Representative postoperative radiographs demonstrating comparable reduction quality between the two groups are shown in Figure 4.

Complications were not significantly different between the two groups. No patients suffered from implant failure (Table 5).

**Table 5:** Complications of the groups studied

Parameter	Group A (n=45)	Group B (n=45)	P	Mean difference or RR (95%CI)
Cut-out of lag screw	3 (6.67%)	1 (2.22%)	0.616	3 (27.77:0.32)
Varus collapse	4 (8.89%)	1 (2.22%)	0.361	4 (0.46:34.41)
Implant failure	0 (0%)	0 (0%)	-	-
Delayed union	0 (0%)	1 (2.22%)	1	-
Superficial infection	1 (2.22%)	2 (4.44%)	1	0.5 (5.32-0.05)
Lateral thigh pain	4 (8.89%)	4 (8.89%)	1	1 (3.75-0.27)

## Discussion

Intertrochanteric (IT) fractures are a significant health concern, particularly among the aging population, and their unstable variants pose a considerable challenge to orthopedic surgeons [16-19]. While the use of proximal femoral nails (PFNs) has revolutionized the treatment of these fractures, achieving optimal stability and minimizing complications remains an active area of research [19].

Although existing evidence supporting trochanteric buttress plate augmentation is largely derived from biomechanical studies and observational series, this lack of high-level evidence was precisely the rationale for conducting a prospective randomized controlled trial.

This study investigated the effectiveness of integrating a TBP with PFN for the management of unstable IT fractures.

In the present study, the integration of TBP with PFN (group B) resulted in significantly better functional outcomes at all postoperative intervals compared to standard PFN (group A). This clinical benefit was achieved without a significant increase in major complications, implant failure, non-union, or femoral fractures. However, this enhanced stability and functional improvement came at the cost of a statistically significant increase in operative time and blood loss in the TBP group, although this did not translate into a higher transfusion rate.

The relatively moderate increase in blood loss is consistent with the additional dissection required for plate application. It suggests that while the TBP augmentation is more invasive than standard nailing, it does not constitute an excessive surgical trauma [20].

Our findings are consistent with those reported by Partheeswar et al., who also observed favorable functional outcomes using the PFN + TBP construct in unstable intertrochanteric fractures [20].

When compared to the literature, our operative parameters and outcomes are consistent. Jain et al. reported a mean operative time of 91.86 ± 12.8 min and blood loss of 144.8 ± 3.6 ml, with a mean mHHS of 94.1 ± 7.5, which is comparable to our findings [21]. The studies by Wang et al. and Ganjale et al. further support the concept, demonstrating significant postoperative improvement in mHHS with the combined approach, despite variations in reported blood loss and operative time [22,23]. Ganjale et al. also reported no cases of non-union or implant breakage, reinforcing the safety profile observed in our study [23].

The strengths of this study lie in its prospective, randomized controlled design, which allows for a robust comparison between the two techniques. Additionally, the inclusion of a diverse patient population with different unstable fracture types enhances the generalizability of our findings.

However, the study has limitations. The open-label design could introduce performance bias, though it was unavoidable. The sample size, while sufficient for the primary outcome, may restrict the broad generalizability of the findings. Furthermore, the six-month follow-up duration, while adequate for assessing fracture union and early functional recovery, may not be sufficient to fully evaluate long-term outcomes and potential late complications, such as implant-related issues or avascular necrosis.

This study has several limitations that should be acknowledged. First, the open-label design could introduce performance bias, though it was unavoidable. The sample size, while sufficient for the primary outcome, may restrict the broad generalizability of the findings. Second, the relatively short follow-up period of six months limits the ability to assess long-term functional outcomes, implant survivorship, and late complications such as implant-related failure or avascular necrosis. Therefore, the findings should be interpreted as reflecting early to mid-term outcomes rather than definitive long-term effectiveness.

Third, the preoperative mHHS was not assessed in the acute post-injury setting. Instead, patients were asked to recall their pre-injury functional status during the week prior to the fracture. While this is a pragmatic and widely accepted methodology in hip fracture research when prospective baseline data are unavailable, it introduces the possibility of recall bias. Patients may overestimate or underestimate their pre-fracture function, and this should be considered when interpreting the magnitude of postoperative functional recovery. Importantly, both groups were subject to the same potential bias, and the non-significant difference between groups suggests that recall error was evenly distributed.

Fourth, functional assessment at early postoperative time points (two weeks and one month) may be influenced by postoperative pain, rehabilitation protocols, and weight-bearing restrictions, and thus reflects early recovery rather than stable functional outcome.

Larger multi-center studies with longer follow-up periods are needed to confirm the long-term benefits and safety of this combined fixation technique. Further research is also warranted to compare the cost-effectiveness of this approach with other treatment options for unstable IT fractures.

## Conclusions

The integration of the trochanteric buttress plate with the proximal femoral nail appears to provide improved early functional outcomes in patients with unstable intertrochanteric fractures. However, radiological outcomes, including fracture reduction accuracy and complication rates, were comparable between the two groups. Therefore, while TBP augmentation may offer benefits in functional recovery, it should be considered a promising adjunctive technique rather than a clearly superior method, and further studies with larger cohorts and longer follow-up are required to confirm its overall effectiveness.
